# Draft Genome Sequences of “*Candidatus* Synechococcus spongiarum,” Cyanobacterial Symbionts of the Mediterranean Sponge *Aplysina aerophoba*

**DOI:** 10.1128/genomeA.00268-17

**Published:** 2017-04-27

**Authors:** Beate M. Slaby, Ute Hentschel

**Affiliations:** a Department of Botany II, Julius-von-Sachs Institute for Biological Sciences, University of Würzburg, Würzburg, Germany; b RD3 Marine Microbiology, GEOMAR Helmholtz Centre for Ocean Research Kiel, Kiel, Germany; c Christian-Albrechts-University of Kiel, Kiel, Germany

## Abstract

We report here four draft genome sequences belonging to clade F of the cyanobacterium “*Candidatus* Synechococcus spongiarum” of the marine sponge *Aplysina aerophoba*, which were collected from two nearby locations in the northern Adriatic Sea. The sequences provide the basis for within-clade comparisons between members of this widespread group of cyanobacterial sponge symbionts.

## GENOME ANNOUNCEMENT

The *Synechococcus/Prochlorococcus* clade represents the most dominant cyanobacterial clade in the world’s oceans, as it is responsible for ~20 to 40% of marine chlorophyll biomass and carbon fixation (1). Sponges (phylum *Porifera*) are important components of marine environments due to their immense filter-feeding capacities and consequent impacts upon coastal food webs and biogeochemical cycles. Their associated dense and diverse microbial communities have received considerable research attention, yet much remains unknown about their *in situ* ecological function(s) (2, 3). The cyanobacterial symbionts “*Candidatus* Synechococcus spongiarum” are widely distributed and highly abundant in sponges around the world and show diversity on the clade level correlating to host sponge phylogeny (4, 5). This mutually beneficial association between sponges and cyanobacteria is thought to be one of the oldest microbe-metazoan interactions.

In a previous study, genomes of four clades of “*Ca.* Synechococcus spongiarum” from different sponge species and geographic locations were compared to each other and to free-living relatives, providing insight into the adaptations of the symbionts to their specific sponge host and to sponges in general (6, 7). Members of different clades of “*Ca.* Synechococcus spongiarum,” despite a 16S rRNA gene sequence identity of ~99%, shared only about half of their gene content (7). A comparison between members of the same clade of this sponge symbiont is lacking.

Here, we present four draft genomes that were sequenced in parallel with the previously published “*Ca*. Synechococcus spongiarum” strain 15L of the *Aplysina aerophoba*–specific clade F (7). Two of them—like 15L—were obtained from the *A. aerophoba* specimens collected near Piran, Slovenia (45°31.07′N, 13°34.10′E) on 7 May 2013, and two were from specimens collected near Rovinj, Croatia (45°5.59′N, 13°37.25′E) on 6 May 2013, from a water depth of ~5 to 7 m. As described for 15L, “*Ca.* Synechococcus spongiarum” cells were sorted from freshly prepared sponge-associated prokaryotes by fluorescence-activated cell sorting targeting their phycoerythrin and chlorophyll *a* autofluorescence (7). By sorting multiple cells into one tube, the target species was enriched from samples of both locations (enrichments named bulk10 [Rovinj] and bulk15 [Piran]). Two 2-µL aliquots of each (10D and 10E from bulk10, and 15M and 15N from bulk15) were amplified with the REPLI-g single-cell kit (Qiagen) and sequenced on an Illumina HiSeq platform (150-bp paired-end reads), as previously described (7). This yielded between 33,791,596 (10E) and 46,038,792 (15N) reads. Quality-filtered sequences were assembled with SPAdes version 3.0.0 (8) and automatically decontaminated (9) in the Joint Genome Institute’s (JGI) single-cell pipeline (http://jgi.doe.gov), resulting in assemblies of about 0.5 Mb (10D and 10E) and 1.5 Mb (15M and 15N). Estimated genome completeness ranged from 21% (10E) to 80% (15M) according to JGI’s completeness estimation.

This data set enables the within-clade comparison of “*Ca.* Synechococcus spongiarum” clade F members of the microbial symbiont consortium of *A. aerophoba* sampled at two geographic locations at the same time, providing insight into similarities and differences at the subclade level.

### Accession number(s).

The whole-genome shotgun sequences of “*Ca.* Synechococcus spongiarum” strains 10D, 10E, 15M, and 15N were deposited in DDBJ/ENA/GenBank under the accession numbers MWLG00000000, MWLF00000000, MWLD00000000, and MWLE00000000, respectively. The versions described in this paper are the first versions MWLG01000000, MWLF01000000, MWLD01000000, and MWLE01000000.
